# Molecular docking analysis reveals the functional inhibitory effect of Genistein and Quercetin on TMPRSS2: SARS-COV-2 cell entry facilitator spike protein

**DOI:** 10.1186/s12859-022-04724-9

**Published:** 2022-05-16

**Authors:** Reji Manjunathan, Vijayalakshmi Periyaswami, Kartik Mitra, Arokiaraj Sherlin Rosita, Medha Pandya, Jayaraman Selvaraj, Lokesh Ravi, Nalini Devarajan, Mukesh Doble

**Affiliations:** 1 Department of Genetics, Dr. ALM Post Graduate Institute of Basic Medical Sciences, Taramani Campus, University of Madras, Chennai, Tamil Nadu India; 2 Multi-Disciplinary Research Unit, Chengalpattu Government Medical College and Hospital, Chengalpattu, Tamil Nadu 603001 India; 3 Department of Biotechnology and Bioinformatics, Holy Cross College, Bharathidasan University, Trichy, Tamil Nadu India; 4 Bioengineering and Drug Design Lab, Department of Biotechnology, Indian Institute of Technology Madras, Chennai, Tamil Nadu India; 5 Department of Bioinformatics, Bishop Heber College (Autonomous, Bharathidasan University), Tiruchirapalli, Tamil Nadu India; 6 KPES Science College, Maharaja KrishnakumarSinhiji Bhavnagar University, Gujarat, India; 7 Saveetha Dental College and Hospitals, Saveetha Institute of Medical and Technical Sciences, Chennai, Tamil Nadu India; 8 Department of Botany, St. Josephs College, Bangalore, Karnataka India; 9 Central Research Laboratory, Meenakshi Ammal Dental College, Maduravoyal, Chennai, Tamil Nadu India; 10 Department of Cariology, Saveetha Dental College and Hospital, Chennai, Tamil Nadu 600077 India

**Keywords:** Transmembrane serine protease 2, SARS-Cov-2 coronavirus, Phyto compounds, Bioinformatics tools, Molecular docking, Molecular dynamics

## Abstract

**Background:**

The Transmembrane Serine Protease 2 (TMPRSS2) of human cell plays a significant role in proteolytic cleavage of SARS-Cov-2 coronavirus spike protein and subsequent priming to the receptor ACE2. Approaching TMPRSS2 as a therapeutic target for the inhibition of SARS-Cov-2 infection is highly promising. Hence, in the present study, we docked the binding efficacy of ten naturally available phyto compounds with known anti-viral potential with TMPRSS2. The aim is to identify the best phyto compound with a high functional affinity towards the active site of the TMPRSS2 with the aid of two different docking software. Molecular Dynamic Simulations were performed to analyse the conformational space of the binding pocket of the target protein with selected molecules.

**Results:**

Docking analysis using PyRx version 0.8 along with AutoDockVina reveals that among the screened phyto compounds, Genistein shows the maximum binding affinity towards the hydrophobic substrate-binding site of TMPRSS2 with three hydrogen bonds interaction ( − 7.5 kcal/mol). On the other hand, molecular docking analysis using Schrodinger identified Quercetin as the most potent phyto compound with a maximum binding affinity towards the hydrophilic catalytic site of TMPRSS2 ( − 7.847 kcal/mol) with three hydrogen bonds interaction. The molecular dynamics simulation reveals that the Quercetin-TMPRSS complex is stable until 50 ns and forms stable interaction with the protein ( − 22.37 kcal/mol of MM-PBSA binding free energy). Genistein creates a weak interaction with the loop residues and hence has an unstable binding and exits from the binding pocket.

**Conclusion:**

The compounds, Quercetin and Genistein, can inhibit the TMPRSS2 guided priming of the spike protein. The compounds could reduce the interaction of the host cell with the type I transmembrane glycoprotein to prevent the entry of the virus. The critical finding is that compared to Genistein, Quercetin exhibits higher binding affinity with the catalytic unit of TMPRSS2 and forms a stable complex with the target. Thus, enhancing our innate immunity by consuming foods rich in Quercetin and Genistein or developing a novel drug in the combination of Quercetin and Genistein could be the brilliant choices to prevent SARS-Cov-2 infection when we consider the present chaos associated with vaccines and anti-viral medicines.

**Supplementary Information:**

The online version contains supplementary material available at 10.1186/s12859-022-04724-9.

## Introduction

The dreadful global pandemic of this century caused by Severe Acute Respiratory Syndrome Coronavirus 2 (SARS-COV2) has affected over 170 million people across the world. It has taken the lives of nearly 38 million people in the past 19 months [1]. Though several vaccines are being used, notable disproportions in the vaccine manufacturing rate and world population size of approximately 7.8 billion significantly limit the speed of global vaccine administration. Moreover, developing an effective vaccine with one hundred percent protection against SARS-CoV-2 infection is tricky due to the possible mutation in the spike glycoprotein of coronavirus. Moreover, all the possible mutations supported the virus to emerge as the most virulent strains in the fore coming waves [2]. Mounting studies show that people with excess viral load and other co-morbidities, especially with diabetes and cardiovascular disease (CVD), are at high risk for COVID-19 associated mortalities [3, 4, and 5]. Thus, in the current situation identifying some methods that can either reduce or prevent the colonization and adhesion of the viruses in the lungs of humans or having the ability to increase the human immune response against viral infections would be more acceptable for the prevention of coronavirus infections.

The mechanism underlying COVID-19 viral entry into a host cell is now well understood. The SARS-CoV-2, the enveloped virus with a positive-sense RNA genome, is majorly composed of four structural glycoproteins, namely spike (S), membrane (M), envelope (E), and Nucleoplasmid (Nsp) [6]. Recently it has been shown that the host cell entry of SARS-CoV-2 is reliant on two host proteins, such as Transmembrane Serine Protease 2 (TMPRSS2) and Angiotensin-Converting Enzyme 2 (ACE2) located on the surface of epithelial cells in the target organ [4, 5, and 6]. Meanwhile, all other proteins, including M, E, and Nsp are involved in the viral particle assembly release into the host cell [7, 8]. The TMPRSS2 primes the viral spike (S) protein by cleaving it at two sites and facilitates the fusion of the viral and host membrane for the smoothening of the cellular entry of viral genome with the aid of a terminal carboxypeptidase and type I transmembrane glycoprotein, ACE2 [1]. Thus, focusing on the inhibition of either ACE2 or TMPRSS2's biological functions has become more attractive targets to prevent the viral genome's entry into the host cells. Among the two proteins, inhibition of TMPRSS2 is more convincing and has recently been shown to inhibit SARS–2-S-driven access in lung cells [9]. Therefore, developing therapeutic agents targeting the inhibition of TMPRSS2 function will have a promising impact against the current and emerging coronavirus outbreaks.

Though many natural molecules are identified against the different target proteins of SARS-COV-2, we selected ten most crucial phyto compounds that exist abundantly in the raw diet based on their profound anti-viral properties (Table 1) [6, 10]. To find the best anti-viral compound against SARS-COV-2 infection, we analyzed the binding efficacy of the selected compounds with the core function site of the human TMPRSS2 using 2 different molecular docking software. The three-dimensional model of TMPRSS2 shows three domains such as an N-terminal Low-Density Lipoprotein (LDL)-receptor class A domain (113–148), a Scavenger Receptor Cysteine-Rich (SRCR) domain (153–246), and a C-terminal peptidase S1 Catalytic domain ranging from 256 to 487 amino acids. It has three catalytic residues, such as HIS296, ASP345, and SER441, in the C terminal serine domain. The six amino acid residues that are more important in the active site make-up of TMPRSS2 are the HIS296, ASP345, and SER441, and are located at the catalytic site (catalytic triad). The remaining three are the ASP435, SER460, and GLY462 and are located at the substrate-binding site (Additional file 1: Fig. S1) [11]. The model was considered to investigate the interactions between the TMPRSS2 and the selected anti-viral phyto compounds.

**Table 1:** List of phyto compounds

S.No	Compound name	PubChem ID	Molecular weight (g/mol)	Chemical structure	Natural sources
1	Resveratrol	445,154	228.24		Fruits—Peanuts, Grapes, Blue, and Blackberries
2	Curcumin	969,516	368.4		Plant—Curcuma
3	Quercetin	5,280,343	302.23		Fruits- Apple, Cherry, Tomatoes, Blueberry
4	Berberine	2353	336.4		Fruits—Oregon grape, European barberry
5	Genistein	5,280,961	270.24		Plants—Lupin, Fava beans, soya beans
6	Beta-carotene	5,280,489	536.9		Vegetables—Carrots, Sweet potatoes, Spinach
7	Lutein	5,281,243	568.9		Vegetables and eggs—Broccoli, peas, spinach, and egg yolks
8	Phenethyl Isothiocycanate	16,741	163.24		Cruciferous vegetable—Watercress
9	Benzyl Isothiocyanate	2346	149.21		Vegetable and plant sources- Pilu oil, papaya seeds, and Alliaria *petiolata*
10	Sulforaphane	5350	177.3		Vegetables—Broccoli, Cauliflower, and Cabbage

## Materials and methods

### Homology modelling

The 3D structure of TMPRSS2 was obtained from Brookhaven protein databank (PDB ID: 7 MEQ) [12]. The TMPRSS2 FASTA sequence (accession number: O15393) was extracted from the UniProt database and modelled with Swiss model server using the crystal structure (PDB ID: 7MEQ) as a template because of the presence of some missing loop regions in the resolved crystal structure [13].

### Structure validation

The quality of the TMPRSS2 3D modelled structure was assessed using the PROCHECK Structure Verification Method. The PROCHECK analysis provides a Ramachandran plot of each residue's bond angles (Phi and Psi angles) and confirms the predicted secondary structure's reliability and 3D conformations. Also, a G-Factor score is generated to quantify the error/deviation probability of the predicted structure. The quality of the developed homology model of TMPRSS2 was assessed using the Ramachandran plot [14].

### Molecular docking using PyRx

#### Protein preparation

The modelled protein structure was prepared for docking using AutoDockTools. First, the Gasteiger charges of the side chain amino acids were computed, followed by the addition of polar hydrogens and the merging of the nonpolar hydrogens.

#### Ligand preparation

The chemical structure of the elected phyto compounds was obtained from the PubChem compound database. The chemicals were imported into OpenBabel for 3D format conversion and were saved in mol format for Argus lab. The spatial optimization was carried out with the help of the Argus Lab 4.0.1 software. The Molecular Mechanics (MM) method UFF has been used in ArgusLab with the "Clean Geometry" option for preliminary refining geometries. Hydrogens have been added through the "Add Hydrogens" feature in the edit column of ArgusLab 4.0.1.

#### Molecular docking

The molecular docking has been performed with AutodockVina using PyRx V.0.8 GUI [15, 16]. The 3D modelled protein was imported into PyRx software, which generated a PDBQT file of the protein structure with all polar hydrogens included. All ligand bonds were considered rotatable. The Lamarckian Genetic Algorithm (LGA) was used to perform all calculations by considering rigid-protein and flexible-ligand docking. The binding site on the receptor was described by creating a grid box with the dimensions of Centre X: − 5.99220259237; Centre Y: − 7.10950986462; Centre: 16.4125813637 Å, with a grid spacing of 0.375 Å, and size of X: 19.0610 Å; Y: 21.9221 Å; Z: 29.8639 Å. Following the completion of the docking study, the ligand docked pose with the least binding energy was chosen. In each case, the eight runs with AutoDockVina were executed, and each runner's best pose was saved. The final affinity value was determined by taking the average affinity for the best stances. The binding pattern of docked complexes, hydrogen bond details, and bond length were studied using the discovery studio visualizer.

### Molecular docking using Schrodinger

#### Ligand preparation

The LigPrep (Schrodinger, LLC, NY, USA, 2009) was used to create the conformation structure of the ligands by removing salt, adding hydrogen molecules, and ionizing at pH (7.0 ± 2.0) [17]. Energy minimization has been performed with the support of the OPLS3e force field by using the regular energy capacity of atomic mechanics and RMSD slice of 0.01 Ǻ to create the minimal energy compound isomer.

#### Preparation of protein

The modelled three-dimensional structure of TMPRSS2 was prepared using the Protein preparation wizard panel in the Schrödinger platform. The protein preparation methods involve many stages such as adding of protons, resolving bond orders, optimisation of protonation states and hydrogen-bond networks and conducting protein structure minimization. The hydrogens were added to ensure the structural requirements, and the side chains were optimized either close to the binding cavity or too near the active site or the salt bridges. The hydrogen atoms were added to the structure, most likely in hydroxyl and thiol hydrogen atoms, protonation states, and tautomers of His residue and Chi 'flip' assignment for ASN, GLN, and HIS residues. The optimized structure was minimized with the OPLS-AA force field until the average root mean square deviation of the non-hydrogen atoms reached 0.3 Å [18].

#### Grid-based molecular docking

The docking study was carried out with the Grid-Based Ligand docking method to analyse the interaction of selected phyto compounds with TMPRSS2. The receptor grid is generated to ensure the involvement of other amino acids in the phyto compound's interactions with TMPRSS2. For receptor, a grid box of 30—×—30—×—30 Å3 with a default inner box (10—×—10— ×—10 Å3) was centred on the corresponding ligand (placed on the appropriate ligand. After the grid generation, all of the prepared conformations of the selected compounds have been docked against the binding site utilising 'extra precision' glide docking (Glide XP), which docks compounds freely [19]. The compounds were chosen for further evaluation based on the different docking parameters such as docking score, glide energy, and physical parameters like hydrogen bonding interactions. The Discovery studio visualizer was used as the Visualization tool for docked ligands.

#### Molecular dynamic simulation

The molecular dynamics simulations of Quercetin and Genistein complexes were performed for 50 ns (ns) using NAMDV.2.14 (with NVIDIA CUDA acceleration) [20–22]. The input files for the simulations were prepared using the CHARMM-GUI web server with the CHARMM force field [23, 24]. The ligand topology and parameter files were prepared using the CgenFF program [25]. Cubic periodic boxes with a minimum distance of 2 nm box edges were assigned. The boxes were filled with water (TIP3P model), and the system was neutralized and saturated with 0.15 M NaCl. Then, the energy minimization and equilibration were performed (NVT and NPT for 250 ps each). The equilibrated complexes were subjected to the production run for 50 ns with CHARMM parameters and topologies generated in CHARMM-GUI. Further, the trajectory analysis (RMSD, RMSF, number of hydrogen bonds, and SASA) was performed using VMD [26]. The ligand ensemble cluster analysis and ligand interaction analysis were performed with UCSF Chimera and DS Visualizer [27]. Post-convergence, MM-PBSA binding free energy analysis was performed with 100 snapshots spanning a region of 5 ns using the CaFE binding energy VMD plugin [28].

## Results

### Homology modeling and validation

The quality of the modeled TMPRSS-2 was analyzed using the PROCHECK program for further structural validation. The graphical representation of the target protein's predicted tertiary structure and Ramachandran plot analysis are shown in Additional file 2: Fig. S2. The Ramachandran plot indicates that 86.9% of the residues of the modeled protein were located within the Most Favored Region, 12.1% in Additionally Allowed Region, and 0.7% of the residues in the disallowed region. Thus the data suggested that the predicted model is highly reliable for further analysis.

### Molecular docking analysis using PyRx

**Fig. 1:**
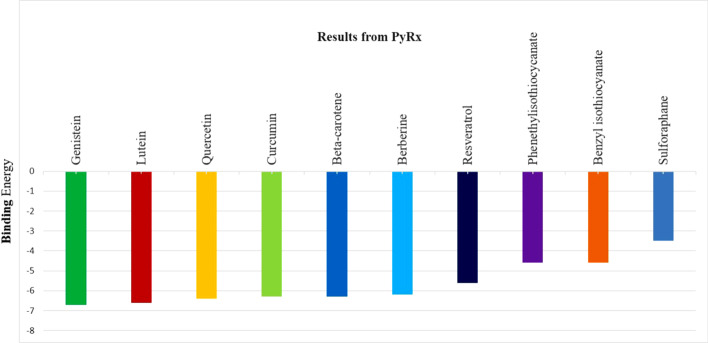
Comparative analysis of binding energy of all compounds obtained from PyRx

**Fig. 2:**
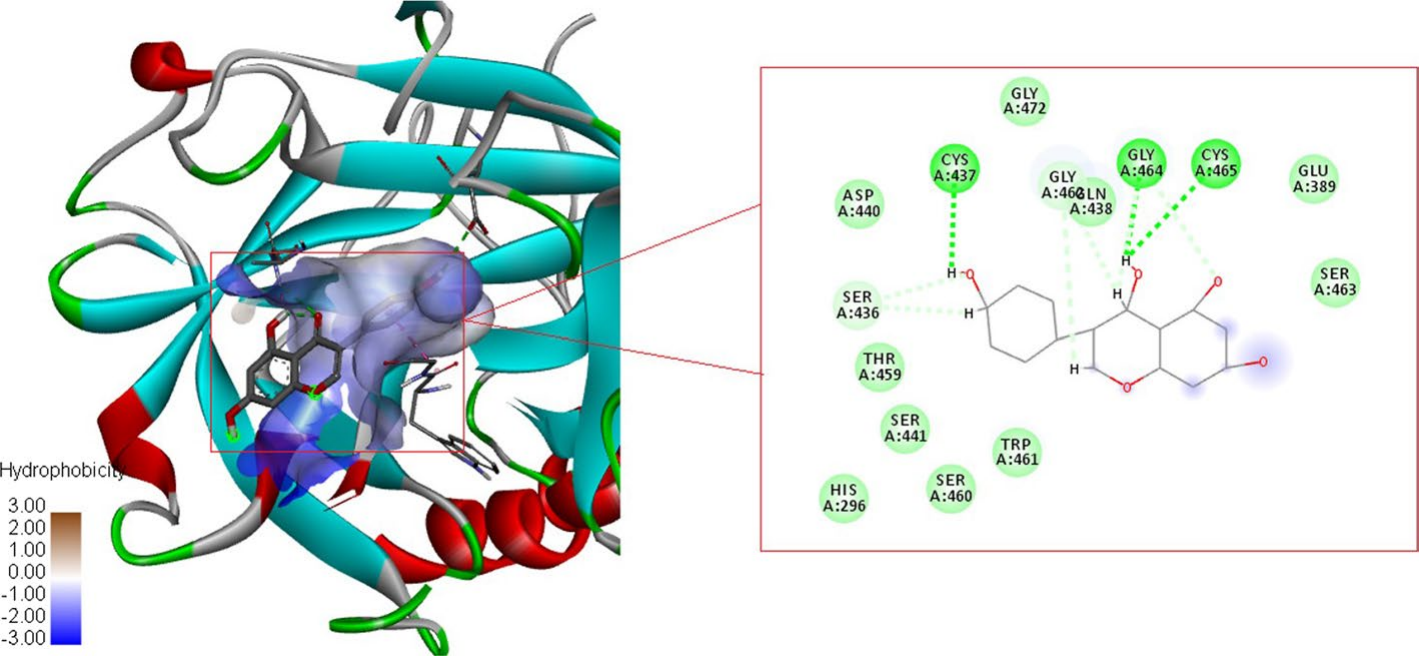
Molecular interaction of TMPRSS2—Genistein (Green colour dotted line—hydrogen bond)

The molecular docking studies were carried out using PyRx to understand the binding affinity of the selected phyto compounds with the modeled TMPRSS2 in terms of measuring the binding energy. The molecular docking studies were carried out in the catalytic site, which comprises the catalytic triad HIS296, ASP345, and SER441, and with the substrate binding site consisting of key substrate interacting residues (ASP435, SER460, and GLY462). Though the catalytic site and the substrate-binding site are positioned adjacent to each other, the hydrophobicity of the pocket was found to be different. The catalytic site was found to be more hydrophilic, consisting of residues such as HIS-274, CYS-281, CYS-297, LEU-302, LYS-342, LYS-392, and CYS-465. In contrast, the substrate-binding site was found to be relatively hydrophobic with amino acids like TRP-461, GLY-472, VAL-473, and TYR-474. The results show that most of the selected phyto compounds exhibit high binding interaction with the catalytic and substrate binding sites of TMPRSS2 protein (Fig. 1). The compounds were ranked based on the binding energy and are listed in Additional file 3: Table S1. The compound Genistein exhibits high binding affinity with TMPRSS2 with a − 6.7 kcal/mol score among the analyzed compounds. The compound could form hydrogen bond interactions with CYS-437, GLY-464 A, and CYS-465 residues located in the substrate-binding site, close to the catalytic site, and thus demonstrated its significant affinity with the catalytic domain of TMPRSS2 (Fig. 2). The rest of the compounds form hydrogen bonds, pi-pi interactions, and other electrostatic interactions with the catalytic sites (HIS296, ASP345, and SER441) and substrate binding sites (ASP435, SER460, and GLY462). All compounds' interaction details and binding energy were shown in the Additional file 3: Table S1 and demonstrated in Additional file 4: Fig. S3.

### Molecular docking using Schrodinger

**Fig. 3:**
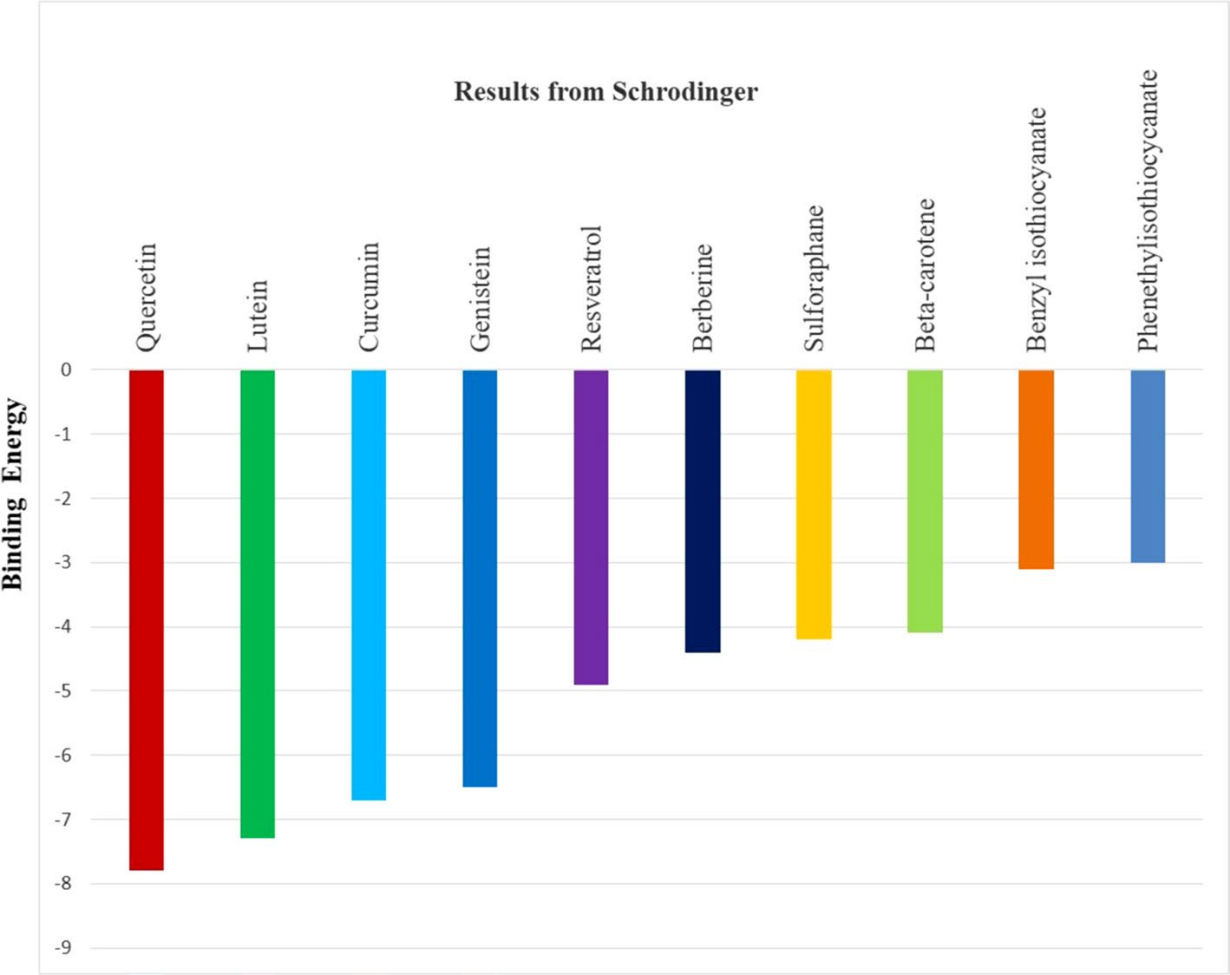
Comparative analysis of binding energy of all compounds obtained from Schrodinger

**Fig. 4:**
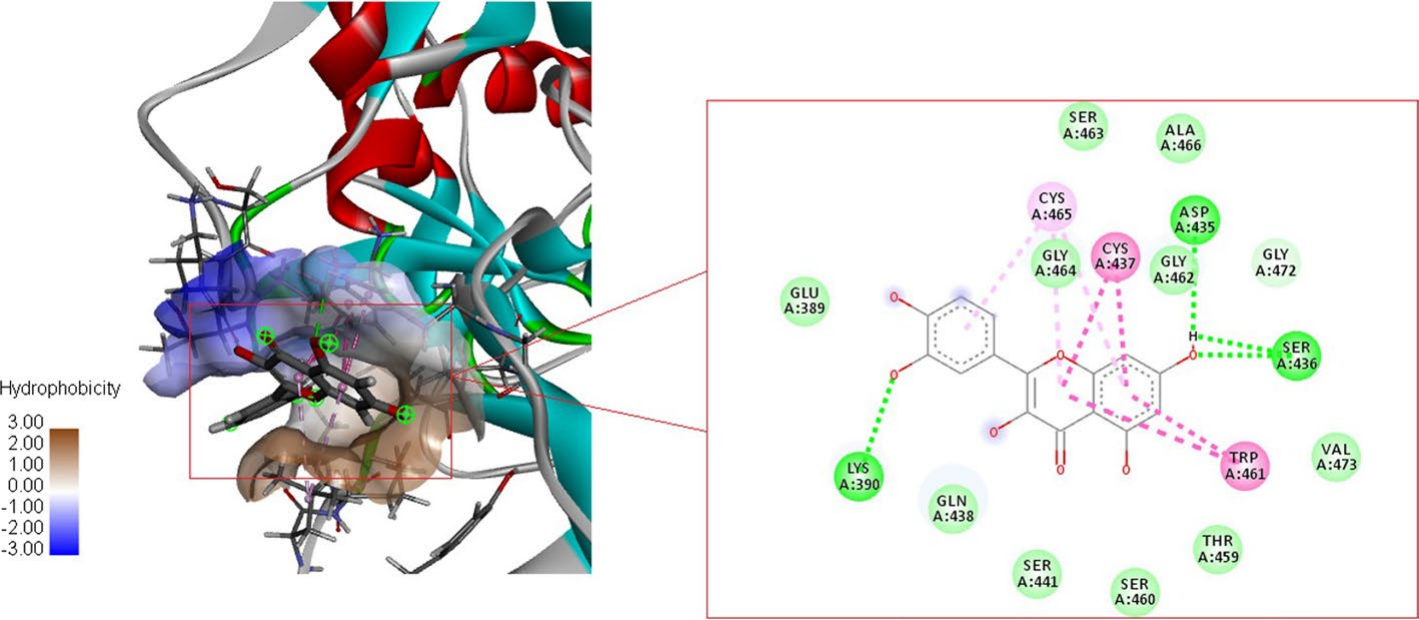
Molecular interaction of TMPRSS2- Quercetin (Green colour dotted line—hydrogen bond; Pink colour—Pi–Pi interaction)

The glide XP docking was performed to confirm the molecular basis of interaction and conformation of the selected phyto compounds to the catalytic and substrate binding sites of TMPRSS2. The details of the molecular docking of all the compounds were enlisted in Supplementary Table 2. Results of XP docking studies indicated that the phyto compounds could exert several potential hydrogen bonds (HB) and non-bonding interactions with the core functional residues of the target protein TMPRSS2. Binding energy analysis of all the ten compounds obtained from the Schrödinger was shown in Fig. 3. The data indicate that compared to other compounds, Quercetin shows the most favorable binding affinity with TMPRSS2 protein and could form strong hydrogen bond interaction with the core functional amino acids such as LYS390, GLN438, SER436, and CYS-465 with a docking score of -7.847 kcal/mol. The compound also exhibits the Pi–Pi interaction with CYS437 and TRP461 amino acids near the catalytic site (Fig. 4). Thus the molecular docking analysis using glide XP indicates that among the analyzed phyto compounds, the Quercetin could fit into the pocket of the serine protease catalytic domain of TMPRSS2 and could competitively inhibit the binding of TMPRSS2 with the viral genome effectively. The detailed interacting residue and all compound’s corresponding binding energy values were listed in Additioanl file 5: Table S2 and Additional file 6: Fig. S4.

### Molecular dynamics simulation

**Fig. 5:**
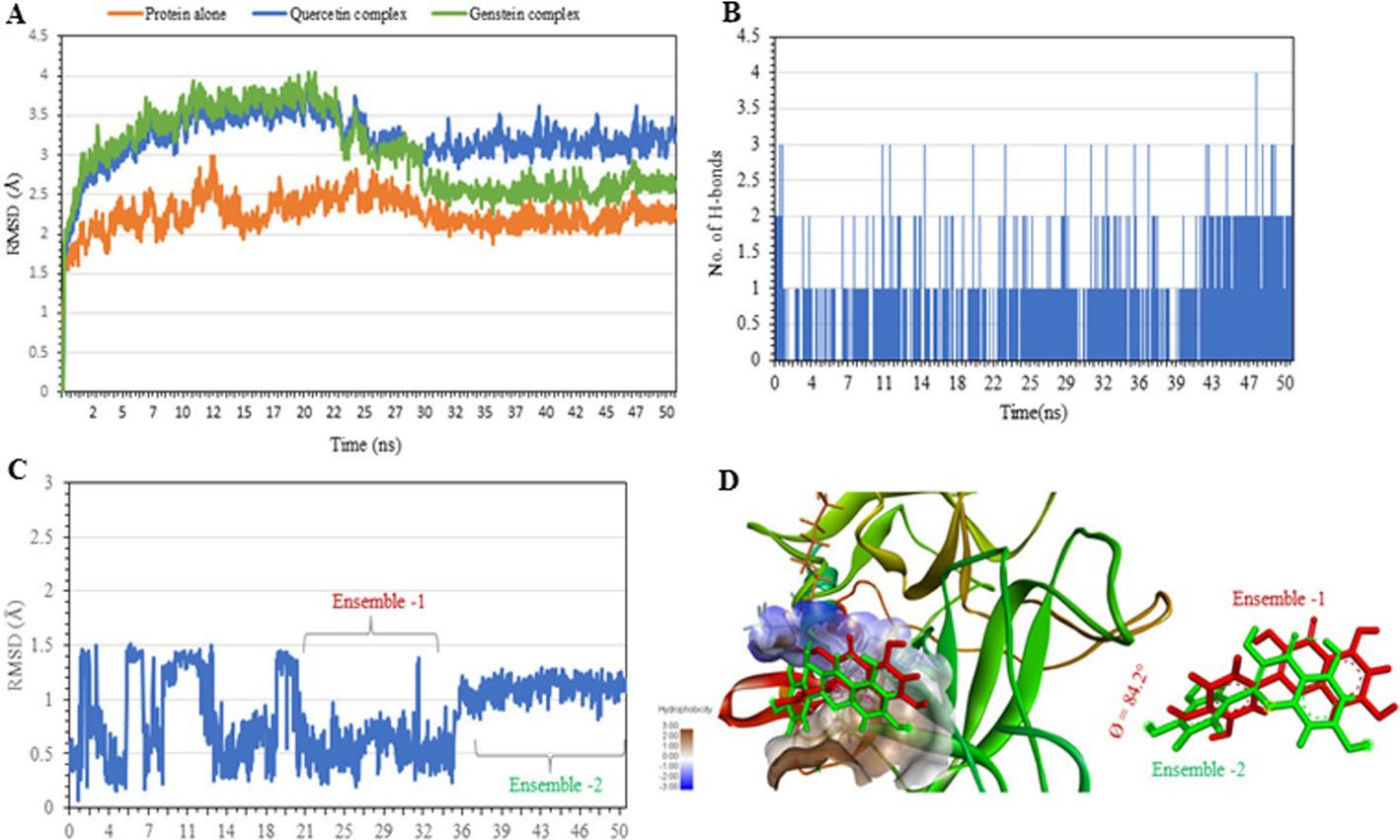
Molecular Dynamic simulation results—**A** Protein backbone RMSD plot of TMPRSS-2 (saffron), Quercetin complex (blue) and Genistein complex (green); **B** The total number of hydrogen bonds of Quercetin complex; **C** Ligand RMSD plot of Quercetin complex; **D** Quercetin complex showing the two different ligand conformation ensemble, ensemble-1 (red, t = 23–32 ns), ensemble-2 (green, t = 33–50 ns)

To assess the binding stability and interaction of Quercetin and Genistein against TMPRSS-2 receptor, the molecular dynamic simulation was performed for 50 ns with NAMDv.2.14 using the CHARMM force field parameters. The protein backbone RMSD of TMPRSS2 alone (Protein alone) and TMPRSS2-Quercetin complex (Quercetin-complex) and TMPRSS2-Genstein complex (Genistein-complex) reached convergence at 30 ns and remained stable till 50 ns (Fig. 5A). Post-convergence, the ligand backbone RMSD of Quercetin was found to show a slight increase at 36 ns by 0.61 Å and continue to remain stable till 50 ns (Fig. 5C). Conformational cluster analysis of Quercetin revealed that the ligand adopted two major conformation ensembles, ensemble-1 (22.6%) and ensemble-2 (54.3%) (Fig. 5D). The structural alignment and ligand interaction analysis showed significant difference between the ensemble-1 and 2 arose due to the orientation of the 3, 4-Dihydroxyphenyl group (side group). In contrast, the orientation and interaction of the core, benzopyran-one ring, was found to remain the same in both the ensembles. In ensemble-1, the side group was protruding outside by interacting with the solvent, whereas in ensemble-2, the side group had re-oriented itself by a rotation of 84.2°around the axis to fit inside the pocket (Fig. 5D). This increased the total number of H-bonds interactions in ensemble-2 concerning ensemble-1 (Fig. 5B).

**Fig. 6:**
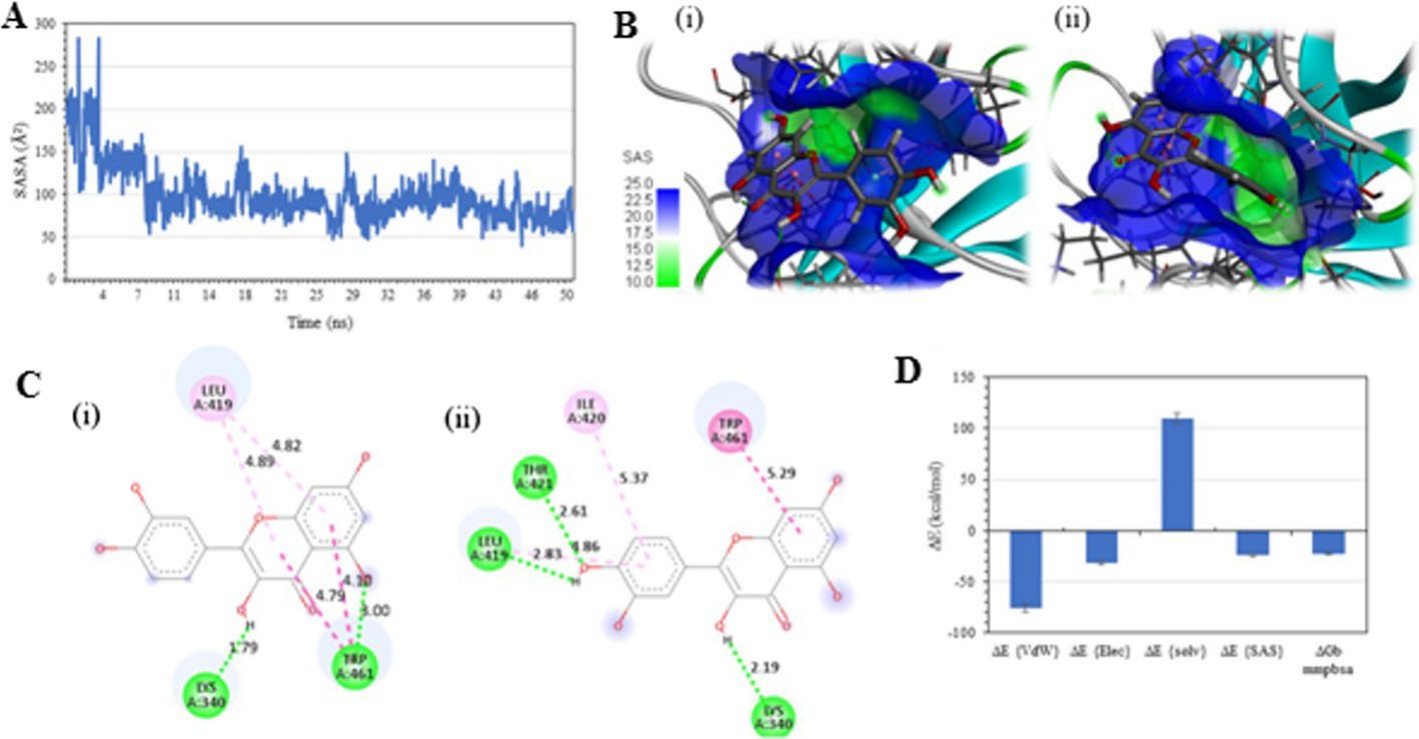
**A** Solvent Accessible Surface Area (SASA) analysis plot of the active site of Quercetin complex; **B** Quercetin complex—showing the decrease in SASA upon re-orientation of a ligand inside the binding pocket; **C** Ligand interaction analysis of Quercetin complex (i) at t = 0 ns and (ii) t = 50 ns; **D** MM-PBSA energy analysis of Quercetin complex

The binding pocket's solvent-accessible surface area (SASA) plot shows that the SASA decreases over time as the ligand fits more stably inside the binding pocket (Fig. 6A). The ligand interaction analysis plot confirms that at t = 0 ns, the side group of Quercetin is solvent-exposed, and at t = 50 ns the compound has undergone conformational re-arrangement (ensemble 2) to fit inside the binding pocket (Fig. 6. B and 6. C). The critical interacting amino acids were LYS340, THR421, LEU419, ILE420, and TRP461 with b-occupancy of 21.3%, 4.2%, 12.7%, 6.2%, and 7.8%, respectively. TRP461 and ILE420 were found to be involved in the hydrophobic and pi-pi stacking interaction. The MM-PBSA binding free energy calculated with the CaFE VMD plugin was found to be with -22.37 kcal/mol, with major energy contribution from Vander Waal (ΔE_{VdW}_) and electrostatic energies (ΔE_{Elec}_) (Fig. 6.D). Moreover, Quercetin bound closer towards the hydrophilic catalytic site of TMPRSS2.

In the case of the Genistein complex, it was observed that the ligand had exited the binding pocket after 26 ns which reflected in an abnormal deviation in the ligand backbone RMSD of Genistein. This may be due to the weak interaction formed by the Genistein with the hydrophobic substrate-binding site of TMPRSS2. To further understand this phenomenon, the iMODS webserver has been used to understand the deformable and highly mobile residues/regions of the Genistein complex. The main-chain deformability is the measure of the capability of a given molecule to deform at each of its residues under dynamic conditions. The iMODS analysis of the Genistein complex revealed that the vital interacting residues of Genistein (CYS465, GLY-465, GLY-462, and CYS-437) have high deformability (Additional file 7: Fig. S5). This deformability may be due to the presence of these residues in the loop region. In addition, the major portion of the benzopyran-4-one core of Genistein was exposed to the solvent and is not involved in the interaction with the protein. The weak interaction of Genistein with the loop residues might be one reason for its unstable binding against TMPRSS2. Moreover, Genistein was bound closer to the Site S2- substrate binding site, which is relatively more hydrophobic in nature. Whereas, Quercetin was bound closer to Site S1, which is hydrophilic (Additional file 8: Fig. S6). Thus, the molecular dynamics study reveals that the compound Quercetin can be a lead molecule with more stable ligand-binding towards TMPRSS2 than Genistein and hence can be taken further to design a novel drug against the current corona virus infection.

## Discussion

The immediate hit of the second corona wave in developing countries such as India has increased the rate of COVID-19 associated mortalities and co-morbidities and more than 2,10,000 deaths within three months [29, 30]. Despite the rapid and massive spread of the SARS-COV2 virus across the world, no specific anti-viral drugs or completely effective vaccines have been developed to date to overcome this global pandemic situation. Moreover, the world is threatened by many waves of SARS-CoV-2 hit with much more genetic variations in the viral RNA structure. In this scenario, developing or boosting the body's self-defence system against viral attack is the right choice to prevent SARS-CoV-2 viral infections [31]. Here we performed a computational approach to identify potential anti-viral phyto molecules that could inhibit the SARS-CoV-2 entry to the host cells. A handful of recent studies have suggested many potential anti-SARS-CoV-2 molecules based on computational results [32, 33, and 34].

It was noted that the SARS-CoV-2 virus uses the ACE2 receptor for cell entry in synergy with the host's TMPRSS2 [35]. Access to the cytosol portion of the host genome is accomplished by acid-dependent proteolytic cleavage of TMPRSS2. It could efficiently activate the S protein to induce fusion of the virus with the host cell membrane for viral activation [10]. Thus, TMPRSS2 represents an essential host factor for SARS-CoV-2 pathogenicity [35]. Hence, developing therapeutic agents targeting TMPRSS2 could be a good measure against the current emerging SARS-CoV-2 outbreak.

The TMPRSS2 possesses a catalytic domain at its C-terminus, typical of chymotrypsin family serine proteases [34]. The TMPRSS2 C-terminal peptidase S1 domain is anticipated to interact with the SARS-CoV-2 spike protein. The amino acids located at the triad of the catalytically active site (HIS296, ASP345, and SER441), and substrate binding sites (ASP435, SER460, and GLY462) are more critical for SARS-CoV-2 spike protein entry into the host cells [33]. Thus, the compounds that could bind with the mentioned core functional unit of TMPRSS2 can potentially restrict the viral access to the host cell. The molecular docking analysis supported to identify the best anti-viral phyto compounds with potential binding affinity with the substrate-binding site of TMPRSS2 protein among the chosen. Based on the scoring parameters and interaction, both Genistein (PyRx) and Quercetin (Schrödinger) could potentially interact with the substrate binding and catalytic site amino acid residues of the TMPRSS2. In addition, the compounds also exhibited interaction with several neighboring residues of the catalytic site and highlighted the importance of neighboring residues in the establishment of the molecular complex between TMPRSS2-Genistein and TMPRSS2-Quercetin complexes [11]. Apart from this, the identification of charged residues at the intermolecular interactions and calculation of binding affinity values supported the complex's structural conformation reliability.

Quercetin (C_15_H_10_O_7_) is one of the most abundant dietary flavonoids found in many fruits, vegetables, leaves, grains, seeds, and red onions and is noted with enriched anti-oxidant properties. Mounting studies have proven the therapeutic effects of Quercetin against various dreadful diseases, including diabetes, cancer, cardiovascular diseases, etc. [35, 36]. Most importantly, many in vitro and in vivo studies have supported potent anti-viral properties of Quercetin [11, 37, 38]. The phytoestrogen Genistein (C_15_H_10_O_5_) is a natural isoflavaone found abundantly in several plants, including lupin, fava beans, soybeans, kudzu, Psoralea, etc. Studies have shown that Genistein possesses enormous anti-viral, anti-oxidant, and anthelmintic properties [39, 40]. Like Quercetin, Genistein is also proven to be an ideal drug to treat various dreadful viral infections, including coronaviridae, HIV, Epsein-Barr virus, herpes simplex virus etc. [38, 40]. Our in *silico* analysis clearly shows that both Quercetin and Genistein could bind to the catalytic and substrate binding sites of TMPRSS2. Quercetin has a higher binding affinity with a -7.847 kcal/mol binding energy towards the catalytic site, which is more hydrophilic than the substrate-binding site. On the contrary, Genistein showed higher binding energy with the substrate-binding site. This may be because the compound Quercetin (XLogP = 1.5) is more hydrophilic than Genistein (XLogP = 2.7). Overall, our research data show that both the phyto compounds inhibit the principal function of the TMPRSS2 protein and thus prevent the fusion of the SARS-CoV-2 viral genome into the host cell.

We also performed a molecular dynamics simulation to understand the binding affinity of Genistein and Quercetin with TMPRSS-2. The molecular dynamics simulation reveals that the Quercetin-TMPRSS complex is stable until 50 ns and the ligand forms a stable interaction with the protein, whereas the Genistein-TMPRSS2 complex was found to suffer unstable binding. It was observed that the Quercetin binds to the S1-hydrophilic site with average MMPBSA binding free energy − 22.37 kcal/mol (at 50 ns) with significant energy contribution from Vander Waal and electrostatic energies. The binding of Quercetin with TMPRSS-2 was also found to decrease the solvent-accessible surface area, indicating a better fit of molecule in the binding pocket. On the other hand, Genistein was found to bind at the S2-hydrophobic site, forming weak interactions with the loop residues and suffering unstable binding, thereby exiting from the binding pocket. This suggests that the catalytic-S1 site of TMPRSS-2, including the residues LYS340, THR421, LEU419, ILE420, and TRP461, can be a potential site for targeting and designing inhibitors. On the contrary, the ligand interaction with loop region residues in the S2-site (substrate binding site), CYS465, GLY465, GLY462, and CYS437, should be avoided to have stable binding. Therefore, Quercetin which has stable and higher binding affinity with S1-site, compared to Genistein, can be considered as a potential molecule for lead optimization and drug development. The ability of Quercetin to exhibit a strong binding affinity with the core functional unit of TMPRSS2 indicates that the compound can effectively inhibit the binding of the virus with the host TMPRSS2 receptor and thereby prevent the viral genome entry into the host cells.

## Conclusion

In summary, viruses pose a danger among the many infectious threats that people face nowadays. Considering all the possibilities of today's pandemic scenario, depending on naturally occurring substances continue to be one of the primary sources of preventing the infectious rate of SARS-CoV-2. Centuries ago, it was proved that a healthy food intake positively influences a person's health, and instead of pills, functional foods can be consumed as part of a regular diet. Improving our natural immunity against SARS-CoV-2 cell entry through consuming healthy foods rich in phyto compounds, especially with anti-viral activities, is the safest and easier way to fight against all kinds of coronavirus infection, including the present global pandemic SARS- CoV-2 spread. In the present study, the molecular docking analysis prioritized Quercetin and Genistein as the best phyto compounds against SARS-Cov-2 infection based on the interaction energy with the binding site of TMPRSS2. But when compared to the Genistein ability, the compound Quercetin has the strongest binding affinity with the core functional unit of TMPRSS2. Hence, the analysis concludes that the phyto compound Quercetin can be used as an effective lead molecule to control the novel coronavirus-2 entry into the human cells. Further studies have to be carried out to prove the molecule's efficacy before setting a therapeutic application target on the same axis.

## Supplementary Information

**Additional file1**: **Figure S1.** Represents the catalytic site (Pink Colour) and substrate binding site (Blue colour) of TMPRSS2.

**Additional file2**: **Figure S2.** Homology model of protein drug target; & validation of predicted homology model.

**Additional file3**: **Table S1.** Molecular docking results for phyto compounds- from PyRx.

**Additional file4**: **Figure S3.** Molecular interaction of TMPRSS2 with phyto compounds obtained from PyRx.

**Additional file5**: **Table S2.** Molecular docking results for phyto compounds -from Schrodinger.

**Additional file6**: **Figure S4.** Molecular interaction of TMPRSS2 with compounds obtained from Schrodinger.

**Additional file7**: **Figure S5.** A) Residue c-alpha deformability analysis of Genistein complex using iMODS. The plot shows the deformability measure of each residue of the Genistein-TMPRSS-2 complex. It can be seen that the deformability measure of GLY464, CYS-465 are high; B) (i) Genistein bound to the loop residues (GLY464, CYS-465, and CYS-437) of TMPRSS-, (ii) Genistein ligand interaction plot with TMPRSS-2.

**Additional file8**: **Figure S6.** The figure shows the binding site of Genistein (ligand in black) and Quercetin (ligand in red) with TMPRSS2. The binding site surface mesh was coloured based on the hydrophobicity index. Genistein was bound closer to the site S2-substrate binding site, which is relatively more hydrophobic than the S1 region. Quercetin was bound closer to towards site S1 catalytic region, which is hydrophilic.

## Data Availability

All data generated or analyzed during this study are included in this published article. The data will be provided on request, rejimanjunath@gmail.com.
