# Supplementary figures and images for: Molecular docking analysis reveals the functional inhibitory effect of Genistein and Quercetin on TMPRSS2: SARS-COV-2 cell entry facilitator spike protein
Source: BMC Bioinformatics. 2022 May 16;23:180. doi: 10.1186/s12859-022-04724-9 (PMC9108711; doi:10.1186/s12859-022-04724-9)

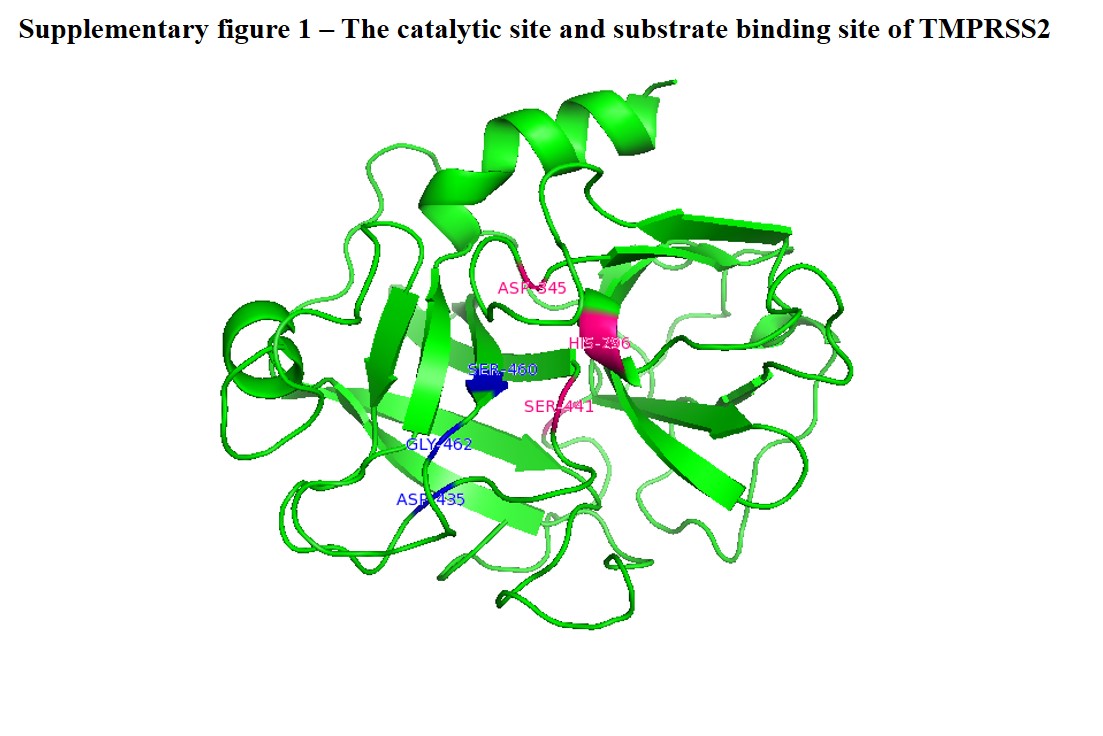

Supplement: Supplementary file 1 — Additional file1: Figure S1. Represents the catalytic site (Pink Colour) and substrate binding site (Blue colour) of TMPRSS2. [file 12859_2022_4724_MOESM1_ESM.jpg]

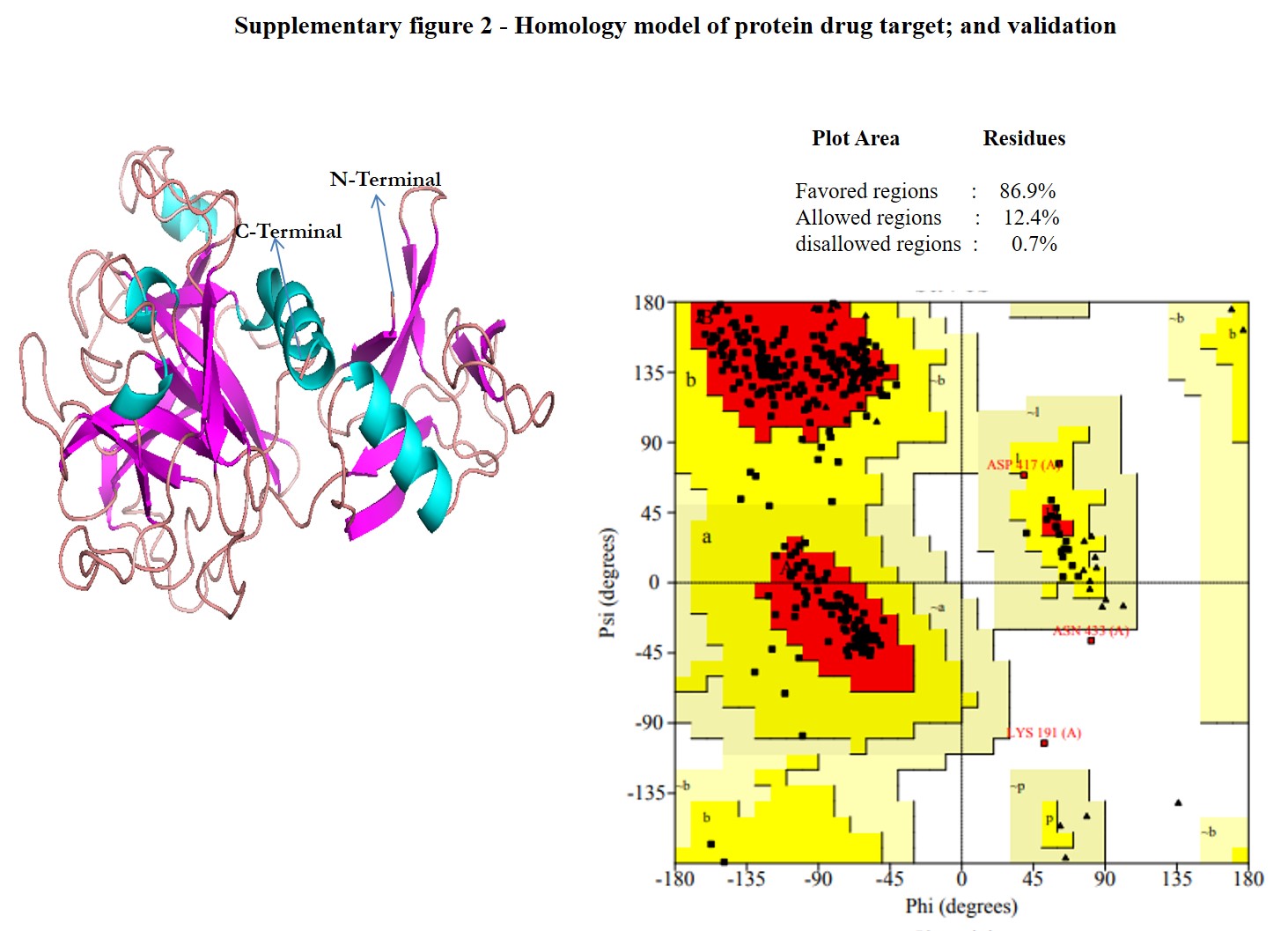

Supplement: Supplementary file 2 — Additional file2: Figure S2. Homology model of protein drug target; & validation of predicted homology model. [file 12859_2022_4724_MOESM2_ESM.jpg]

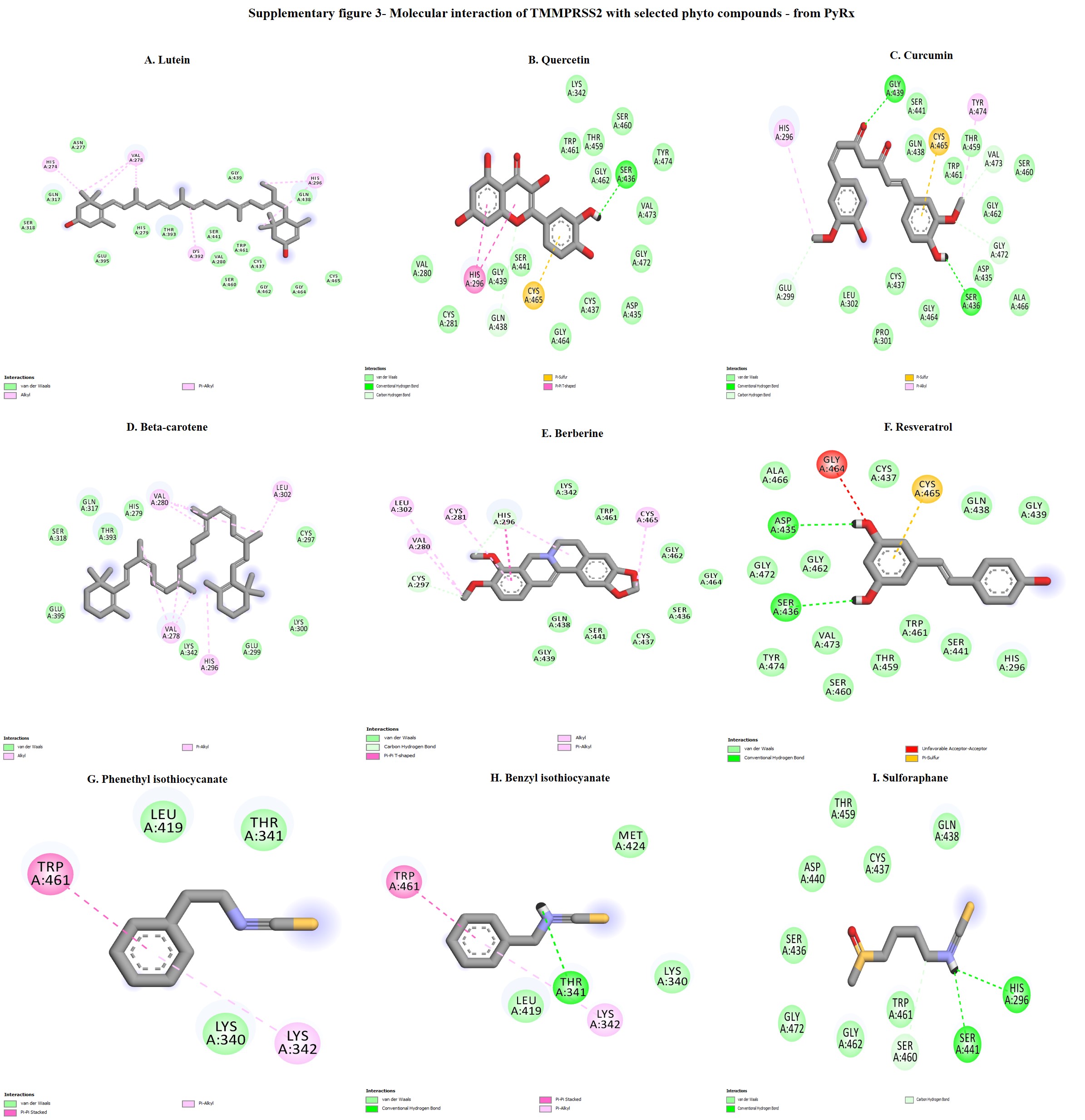

Supplement: Supplementary file 4 — Additional file4: Figure S3. Molecular interaction of TMPRSS2 with phyto compounds obtained from PyRx. [file 12859_2022_4724_MOESM4_ESM.jpg]

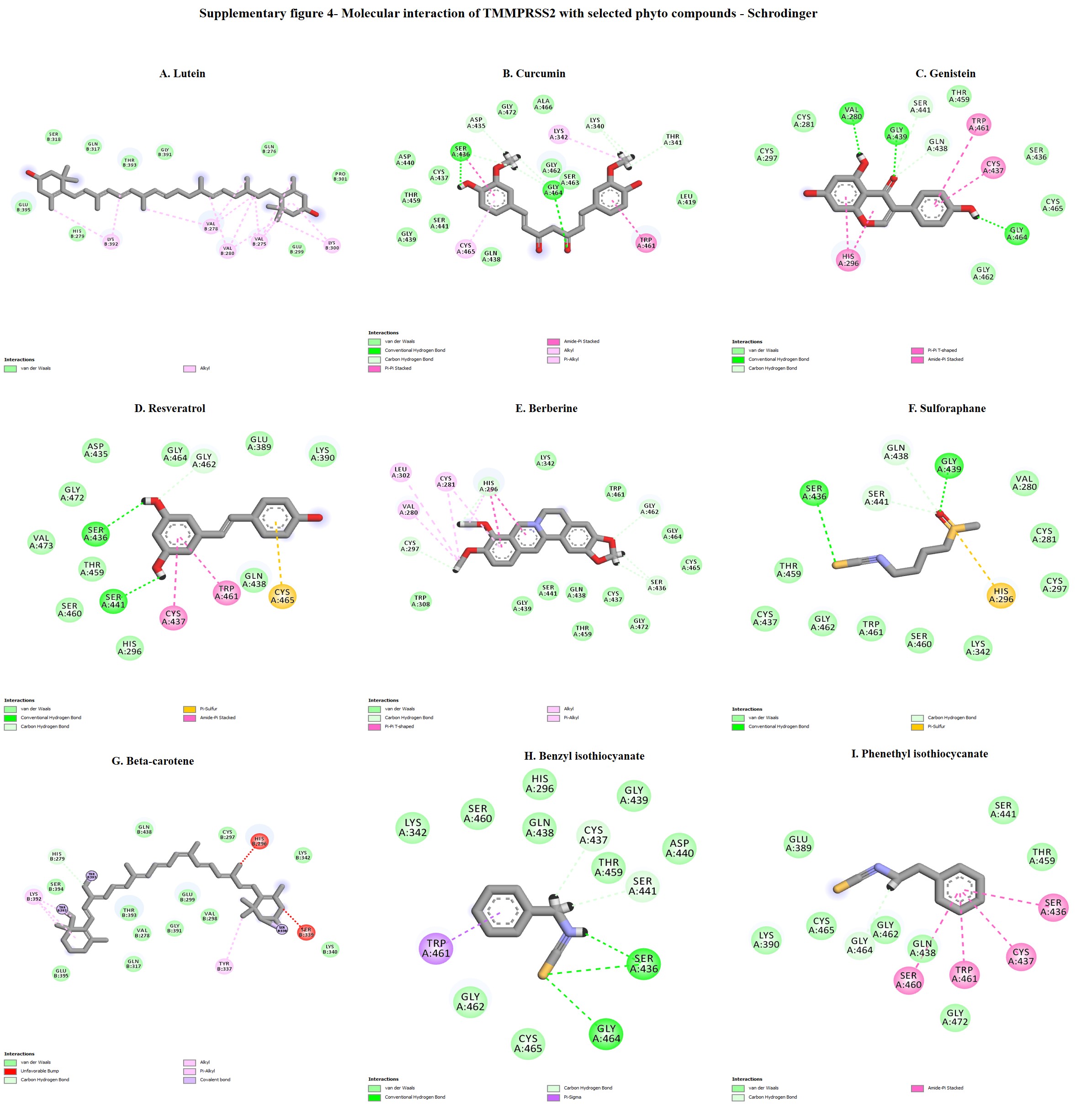

Supplement: Supplementary file 6 — Additional file6: Figure S4. Molecular interaction of TMPRSS2 with compounds obtained from Schrodinger. [file 12859_2022_4724_MOESM6_ESM.jpg]

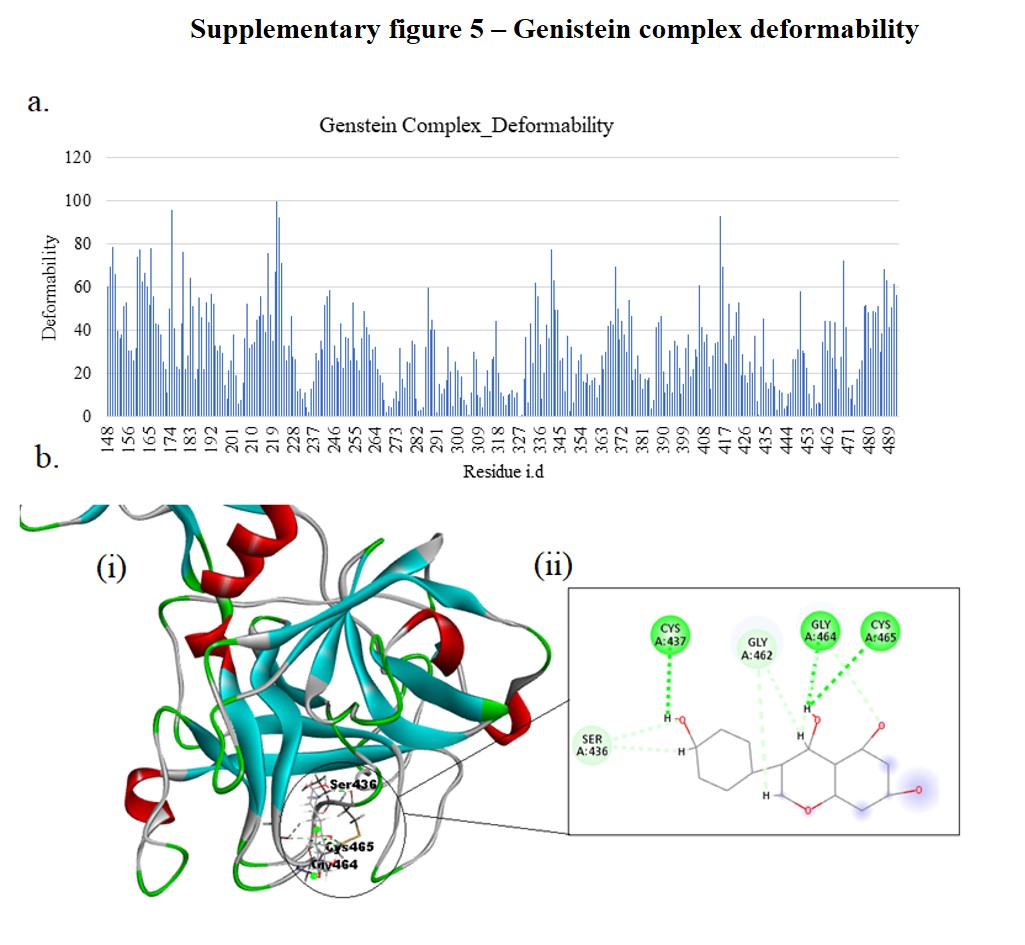

Supplement: Supplementary file 7 — Additional file7: Figure S5. A) Residue c-alpha deformability analysis of Genistein complex using iMODS. The plot shows the deformability measure of each residue of the Genistein-TMPRSS-2 complex. It can be seen that the deformability measure of GLY464, CYS-465 are high; B) (i) Genistein bound to the loop residues (GLY464, CYS-465, and CYS-437) of TMPRSS-, (ii) Genistein ligand interaction plot with TMPRSS-2. [file 12859_2022_4724_MOESM7_ESM.jpg]

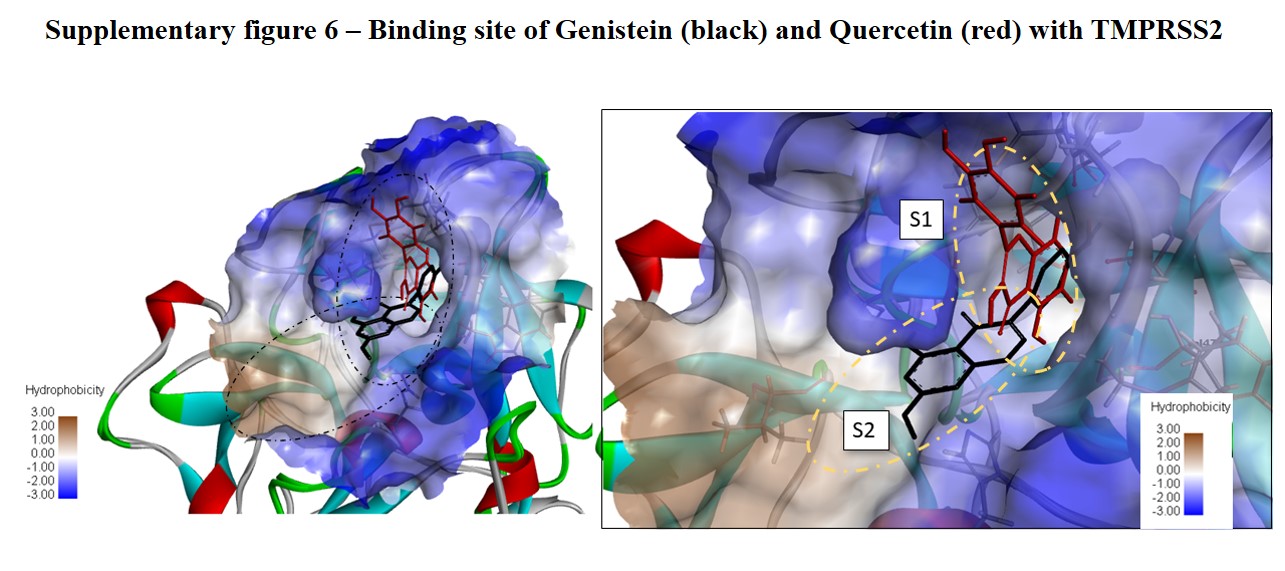

Supplement: Supplementary file 8 — Additional file8: Figure S6. The figure shows the binding site of Genistein (ligand in black) and Quercetin (ligand in red) with TMPRSS2. The binding site surface mesh was coloured based on the hydrophobicity index. Genistein was bound closer to the site S2-substrate binding site, which is relatively more hydrophobic than the S1 region. Quercetin was bound closer to towards site S1 catalytic region, which is hydrophilic. [file 12859_2022_4724_MOESM8_ESM.jpg]
