# Supplementary material for: Molecular docking analysis reveals the functional inhibitory effect of Genistein and Quercetin on TMPRSS2: SARS-COV-2 cell entry facilitator spike protein
Source: BMC Bioinformatics. 2022 May 16;23:180. doi: 10.1186/s12859-022-04724-9 (PMC9108711; doi:10.1186/s12859-022-04724-9)
Supplement: Supplementary file 3 — Additional file3: Table S1. Molecular docking results for phyto compounds- from PyRx. [file 12859_2022_4724_MOESM3_ESM.docx]

| **S.No** | **Compound Name** | **Pubchem id** | **Docking Score**  **Kcal/mol** | **Hydrogen Bond interaction** | **Other interactions** |
| --- | --- | --- | --- | --- | --- |
| **1** | Genistein | 5280961 | -6.7 | CYS-437  GLY-464  CYS-465 | -- |
| 2 | Lutein | 5281243 | -6.6 | - | HIS-274( Pi-alkayl  VAL-278( Pi-alkayl)  LYS-392( Pi-alkayl)  HIS-296( Pi-alkayl) |
| 3 | Quercetin | 5280343 | -6.4 | SER-436 | HIS-296 (Pi-Pi)  CYS-465(Pi-Sulfur) |
| 4 | Curcumin | 969516 | -6.3 | SER-436  GLY-439 | HIS-296(Pi- alkayl )  CYS-465(Pi-Sulfur)  GLY-472(Vander wals)  VAL-473(Vander wals)  TYR-474( Pi- alkayl) |
| 5 | Beta-carotene | 5280489 | -6.3 | - | VAL-278( Pi-alkayl  VAL-280( Pi-alkayl)  HIS-296(Pi-alkayl)  LEU-302( Pi-alkayl) |
| 6 | Berberine | 2353 | -6.2 | - | CYS-297 (Vander wals)  HIS-296 (Vander wals)  CYS-281(Pi-alkayl)  VAL-280(Pi-alkayl)  LEU-302 (Pi-alkayl)  CYS-465( Pi-Sulfur) |
| 7 | Resveratrol | 445154 | -5.6 | ASP-435  SER-436 | CYS-465( Pi-Sulfur) |
| 8 | Phenethylisothiocycanate | 16741 | -4.6 | - | LYS-342( Pi-alkayl)  TRP-461((Pi-Pi) |
| 9 | Benzyl isothiocyanate | 2346 | -4.6 | THR-341 | TRP-461((Pi-Pi)  LYS-342(Pi-alkayl) |
| 10 | Sulforaphane | 5350 | -3.5 | HIS-296  SER-441 | - |

**Supplementary Table 1: Molecular docking results for phyto compounds- from PyRx**
