# Supplementary material for: Molecular docking analysis reveals the functional inhibitory effect of Genistein and Quercetin on TMPRSS2: SARS-COV-2 cell entry facilitator spike protein
Source: BMC Bioinformatics. 2022 May 16;23:180. doi: 10.1186/s12859-022-04724-9 (PMC9108711; doi:10.1186/s12859-022-04724-9)
Supplement: Supplementary file 5 — Additional file5: Table S2. Molecular docking results for phyto compounds -from Schrodinger. [file 12859_2022_4724_MOESM5_ESM.docx]

| **S.No** | **Compound Name** | **Pubchem id** | **Docking Score kcal/mol** | **Hydrogen bond interaction** | **Other interactions** |
| --- | --- | --- | --- | --- | --- |
| **1** | Quercetin | 5280343 | -7.847 | LYS-390,  ASP-435  SER-436 | TRP-461(Pi-Pi)  CYS-437(Pi-Pi)  CYS-465(Pi-pi) |
| **2** | Lutein | 5281243 | -7.3 | **-** | VAL-275(Pi-Akyl)  VAL-278( Pi-Akyl)  VAL-280( Pi-Akyl)  LYS-300( Pi-Akyl)  LYS-392( Pi-Akyl)) |
| 3 | Curcumin | 969516 | -6.707 | SER-436  GLY-464 | LYS-340(Vander Waals)  THR-341(Vander Waals)  LYS-342(Pi-Akyl)  ASP-435( Vander Waals)  TRP-461(Pi-Pi)  CYS-465(Pi-Akyl) |
| 4 | Genistein | 5280961 | -6.591 | VAL-280  GLY-439  GLY-464 | HIS-296((Pi-Pi)  CYS-437(Pi-Pi)  TRP-461(Pi-Pi)  SER-441(Vander Waals)  GLN-438( Vander Waals) |
| 5 | Resveratrol | 445154 | -4.969 | SER-436  SER-441 | CYS-465(Pi-sulfur)  CYS-437(Amide-Pi)  TRP-461( Amide-Pi)  GLY-462(Vander Waals) |
| 6 | Berberine | 2353 | -4.431 | - | VAL-280 (Pi-Alkyl  CYS-281( Pi-Alkyl  LEU-302(Pi-Alkyl)  HIS-296(Pi-Pi) |
| 7 | Sulforaphane | 5350 | -4.206 | SER-436  GLY-439 | HIS-296(Pi-sulfur)  GLN-438(Vander Waals)  SER-441(Vander Waals) |
| 8 | Beta-carotene | 5280489 | -4.140 | - | HIS-279  TYR-337(Pi-Alkyl)  LYS-392(Pi-Alkyl) |
| 9 | Benzyl isothiocyanate | 2346 | -3.199 | SER-436  GLY-464 | CYS-437(Vander Waals)  TRP-461(Pi-sigma) |
| 10 | Phenethylisothiocycanate | 16741 | -3.054 | - | GLY-464(Vander Waals)  SER-460(Amide-Pi)  TRP-461( Amide-Pi)  CYS437(Amide-Pi)  SER-436(Amide-Pi) |

**Supplementary Table 2: Molecular docking results for phyto compounds -from Schrodinger**
